# Novel Ultrahigh-Performance ZnO-Based Varistor Ceramics

**DOI:** 10.1021/acsami.1c07735

**Published:** 2021-07-23

**Authors:** Tian Tian, Liaoying Zheng, Matejka Podlogar, Huarong Zeng, Slavko Bernik, Kunqi Xu, Xuezheng Ruan, Xun Shi, Guorong Li

**Affiliations:** † CAS Key Laboratory of Inorganic Functional Materials and Devices, Shanghai Institute of Ceramics, Chinese Academy of Sciences, Shanghai 201899, China; ‡ State Key Laboratory of High Performance Ceramics and Superfine Microstructure, Shanghai Institute of Ceramics, Chinese Academy of Sciences, Shanghai 200050, China; § Department for Nanostructured Materials, Jozef Stefan Institute, Ljubljana SI-1000, Slovenia

**Keywords:** ZnO, Cr_2_O_3_, varistor ceramics, double-Schottky barriers, microstructure, *I*−*V* characteristics

## Abstract

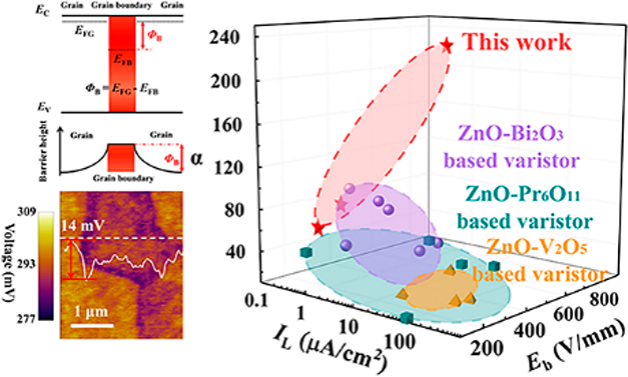

The nonlinear response of a material to an external stimulus is vital in fundamental science and technical applications. The power-law current–voltage relationship of a varistor is one such example. An excellent example of such behavior is the power-law current–voltage relationship exhibited by Bi_2_O_3_-doped ZnO varistor ceramics, which are the cornerstone of commercial varistor materials for overvoltage protection. Here, we report on a sustainable, ZnO-based varistor ceramic, without the volatile Bi_2_O_3_, that is based on Cr_2_O_3_ as the varistor former and oxides of Ca, Co, and Sb as the performance enhancers. The material has an ultrahigh α of up to 219, a low *I*_L_ of less than 0.2 μA/cm^2^, and a high *E*_b_ of up to 925 V/mm, making it superior to state-of-the-art varistor ceramics. The results provide insights into the design of materials with specific characteristics by tailoring states at the grain boundaries. The discovery of this ZnO-Cr_2_O_3_-type varistor ceramic represents a major breakthrough in the field of varistors for overvoltage protection and could drastically affect the world market for overvoltage protection.

## Introduction

**Figure 1:**
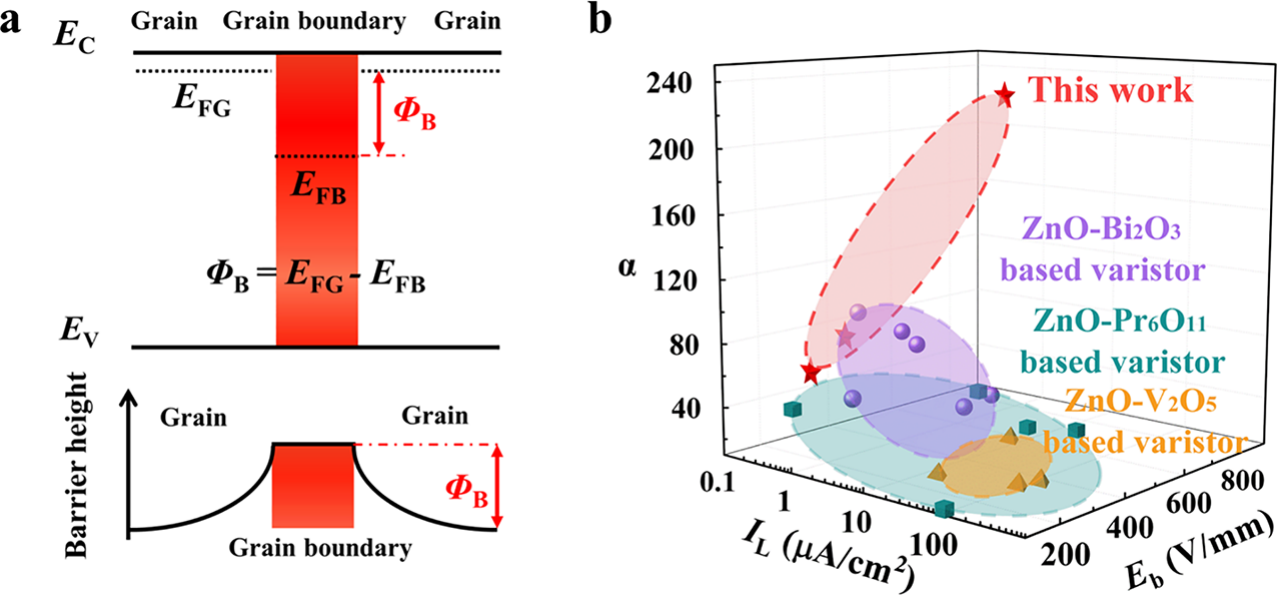
High-performance ZnO-based varistors. (a) Schematic diagram of the double-Schottky barriers (DSB) at the grain boundaries (GBs), where *E*_C_ is the conduction band, *E*_V_ is the valence band, and φ_B_ is the Schottky barrier height, which governs the nonlinear *I–V* characteristics. (b) Nonlinear coefficients (α) vs leakage currents (*I*_L_) and breakdown voltage (*E*_b_) of ZnO-based varistors developed in this work. Literature data of ZnO-based varistors are listed for comparison.^10−12,14−25^

Varistors, better described as *vari*able resi*stors*, are used in billions of low-power electronic devices and heavy-duty electrical-energy-distribution systems to protect circuits from transient voltage surges by means of their nonlinear current–voltage (*I–V*) characteristics.^1−3^ Specifically, a varistor is highly resistive at a low applied voltage but becomes conductive very quickly when the applied voltage exceeds a material-specific threshold known as the breakdown voltage *E*_b_. Above *E*_b_, the nonlinear *I–V* characteristics are empirically described by a power-law function *I* = *bV*^*α*^, where *b* is a constant and α is a nonlinear coefficient. The value of α is thus a gauge of how responsive the varistor is to the transient voltage surge. As illustrated in Figure 1a, the value of α is physically governed by the height of the double-Schottky barrier (DSB), φ_B_, at the grain boundary (GB);^4,5^ φ_B_ = |*E*_FB_ – *E*_FG_|, where *E*_FB_ and *E*_FG_ are the Fermi levels in the GB and the grain, respectively. Besides the high α value, a high-performance varistor entails a small leakage current (*I*_L_), which is the current at 0.75 × *E*_b_. Low values of *I*_L_ ensure a low chance of thermal runaway, good stability and aging behavior, and also a low power consumption.^6^

Among the metal–oxide varistors (MOVs) that are the state-of-the-art varistors, ZnO-based varistors have the highest DSB heights φ_B_ and superior nonlinear properties (high α values and low *I*_L_ values).^6^ Note that pristine ZnO displays a very weak, nonlinear *I–V* characteristic, reflecting *E*_FB_ and *E*_FG_ that are nearly equal. It takes varistor former(s), also known as varistor activator(s), to create defect complexes at the GB, which shift the *E*_FB_ to a deeper position in the band gap, and the resulting φ_B_ gives rise to the DSB and in turn elicits nonlinear *I–V* characteristics in the ZnO.^5^ In parallel with the varistor former(s) implemented at the GB, performance enhancer(s) can be doped into the grains to actively raise the *E*_FG_ and thus increase φ_B_ to higher α and smaller *I*_L_ values.^6−8^

Other than α and *I*_L_, a high *E*_b_ value is desired for surge protection and a reduction of device size in high-voltage dc power transmission and heavy-duty power-supply systems, thereby broadening the scope of applications. However, the increase of *E*_b_ tends to worsen α and *I*_L_ simultaneously.^3,9^ As a result, the state-of-the-art ZnO-based varistors, with varistor formers such as Bi_2_O_3_, Pr_6_O_11_, and V_2_O_5_ along with their corresponding performance enhancers, such as Sb, Co, Mn, Ni, and Cr, typically achieve an α value of less than 60, an *I*_L_ values higher than 1 μA/cm^2^, and an *E*_b_ value in the range 200–500 V/mm.^10−12^

After exploring a large phase space (Supporting Information), here we report on a novel ZnO-based varistor ceramic that uses Cr as the varistor former and Ca, Co, and Sb as the performance enhancers. These ceramics have record-high nonlinear characteristics, manifested in an ultrahigh α value up to 219, a very low *I*_L_ value of less than 0.2 μA/cm^2^, and a high *E*_b_ value of 925 V/mm, making them superior to today’s varistor ceramics. For conciseness, we only list the performance of ZnO-based varistors for comparison in Figure 1b. Moreover, the materials sustainability (volatility and toxicity) of these ultrahigh-performance varistors is significantly improved. Specifically, the compositions avoid the use of highly volatile bismuth oxide,^13^ expensive rare earths like praseodymium, or the toxic vanadium oxide.

## Experimental Section

### Sample Preparation

Reagent-grade powders of ZnO, Cr_2_O_3_, CaCO_3_, Co_3_O_4_, and Sb_2_O_3_ were used for preparation of the materials. Cr-added ZnO with nominal compositions of (1 – *x*) mol ZnO-*x* mol Cr_2_O_3_ (*x* = 0.05%, 0.1%, 0.2%, 0.3%, and 0.4%, namely, ZnO-*x*Cr_2_O_3_), Ca-doped ZnO-0.1% Cr_2_O_3_ (Zn_0.979_Cr_0.002_Ca_0.02_O_1.002_, aka 97.9 mol % ZnO + 0.1 mol % Cr_2_O_3_ + 2 mol % CaCO_3_ in raw materials), (Co, Ca)-codoped ZnO-0.1%Cr_2_O_3_ (Zn_0.974_Cr_0.002_Ca_0.02_Co_0.015_O_1.017_, aka 97.4 mol % ZnO + 0.1 mol % Cr_2_O_3_ + 2 mol % CaCO_3_ + 0.5 mol % Co_3_O_4_ in raw materials), (Co, Ca, Sb)-doped ZnO-0.1% Cr_2_O_3_ (Zn_0.9725_Cr_0.002_Ca_0.02_Co_0.015_Sb_0.003_O_1.02_, aka 97.25 mol % ZnO + 0.1 mol % Cr_2_O_3_ + 2 mol % CaCO_3_ + 0.5 mol % Co_3_O_4_ + 0.15 mol % Sb_2_O_3_ in raw materials), and (Co, Ca)-codoped ZnO (Zn_0.975_Ca_0.02_Co_0.015_O_1.015_, aka 97.5% mol ZnO + 2 mol % CaCO_3_ + 0.5 mol % Co_3_O_4_ in raw materials) ceramics were synthesized by a conventional solid-state technique. We used CaCO_3_ instead of CaO because CaO is not stable in air. At high temperatures, CaCO_3_ was decomposed to CaO and CO_2_ gas and then Ca is doped into ZnO. The starting powders were wet mixed by ball milling for 8 h, followed by drying at about 120 °C for 5 h, calcining at 450 °C for 2 h, and then cold pressed as pellets with a diameter of 12 mm and thickness of 1 mm. Finally, the pellets were sintered at 1200 °C for 3 h. Silver pastes were covered on the sample’s opposite surfaces and then dried at 560 °C for 15 min as electrodes.

### Characterization

X-ray diffraction using Cu Kα radiation (D/max 2550 V, Rigaku, Tokyo, Japan) was used to analyze the phase purity. Microstructural observations were carried out on the polished surfaces using a field-emission scanning electron microscope (Magellan 400, FEI Co., USA). Energy-dispersive X-ray spectroscopy (Oxford Instrument, UK) was performed to detect elements and distributions. Spherical aberration-corrected transmission electron microscopy (HF 5000, Hitachi, Japan) with energy-dispersive X-ray spectroscopy (Oxford X-Max 100TLE, UK) and transmission electron microscopy (Tecnai-F20, FEI Co., USA) were used to observe the microstructures and compositions of the grain boundaries (GBs). The micro-Raman spectra were recorded with a Renishaw InVia Confocal micro-Raman system using the 532 nm line as an excitation source. The *I*–*V* curves were recorded with a high-voltage digital sourcemeter (Keithley 2410, Keithley Instruments Inc., USA). By convention, the breakdown voltage (*E*_b_) was measured at a current of 1 mA/cm^2^, and the leakage current density (*I*_L_) was measured at an electric field of 0.75 × *E*_b_. The nonlinear coefficient (α) was fitted by the equation *I* = *bV*^*α*^ when the voltage is larger than *E*_b_. The dielectric spectra were measured using a broad-band dielectric spectrometer (NovoControl, Hundsangen, Germany) in the frequency range from 0.1 Hz to 1 MHz with an amplitude voltage of 1 V. Ag paste (fired at 560 °C for 15 min) electrodes were used. The dc conductivity (*f* < 1 Hz) was obtained with dielectric spectroscopy at *f* = 0.1. The data from the impedance measurements were analyzed using the commercial software (Z-VIEW, version 3.1). Kelvin probe force microscopy (KPFM) measurements were performed on the atomic force microscopy (AFM) platform to observe the surface potential images. An ac modulation voltage of 2 V at 55 kHz in the lift mode with a distance of approximately 10 nm between the tip and the sample and a conductive Pt/Ir-coated tip (PR-EX-KPFM-5) were used. Additional information is available from the Wiley Online Library or from the author.

## Results and Discussion

**Figure 2:**
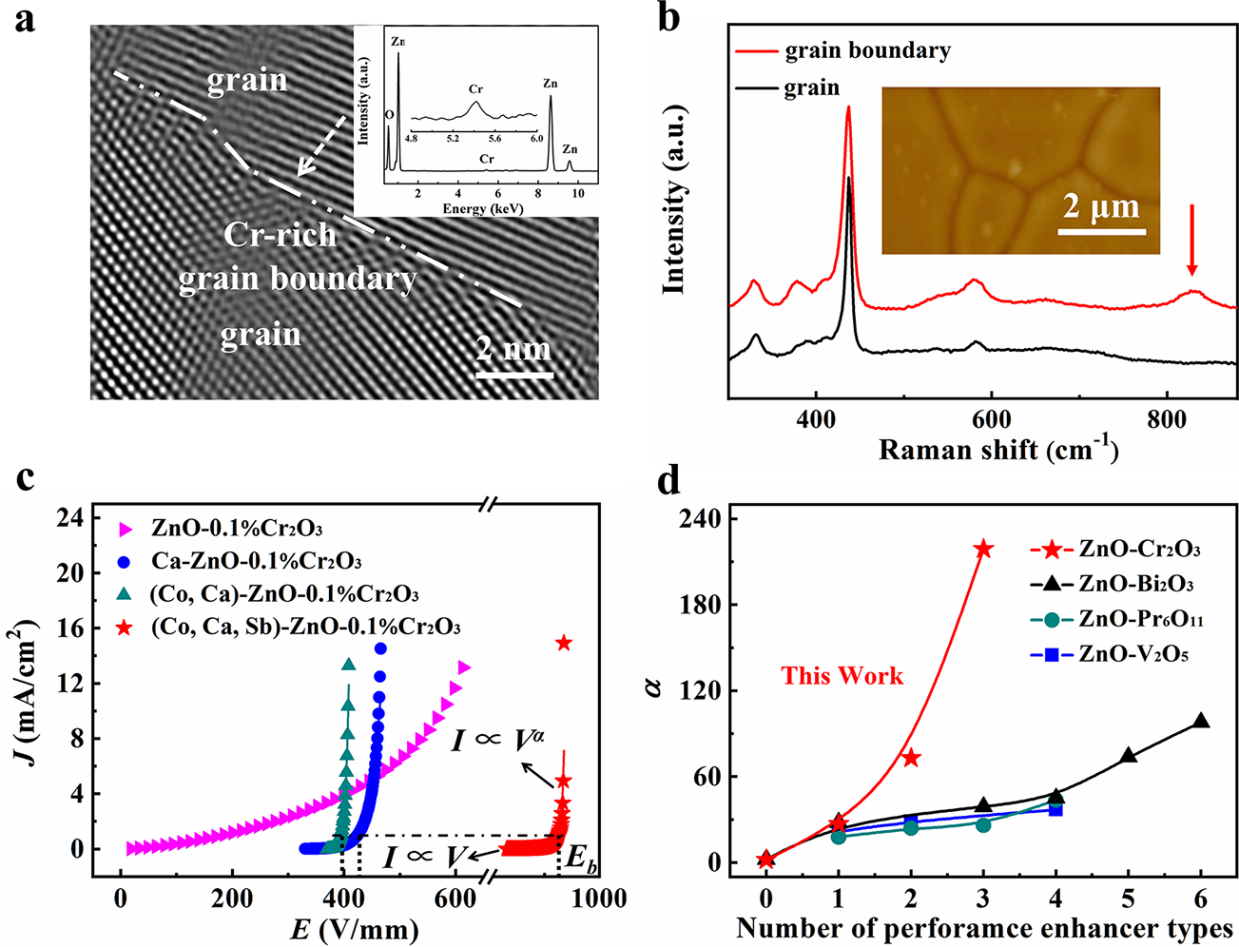
Microstructures and electrical properties of Cr-added ZnO-based varistors. (a) High-resolution transmission electron microscopy (HRTEM) image of the grain boundary (GB) of 0.2% Cr-added ZnO. (Inset) Energy-dispersive X-ray spectroscopy (EDS) analysis of the GB. (b) Micro-Raman spectra of the grain and the GB of the 0.2% Cr-added ZnO. (c) *J–E* characteristics of the Cr-added ZnO varistors with different performance enhancers. Solid lines are the fitting results using the equation *I* = *bV*^*α*^. (d) Nonlinear coefficient α against the number of performance enhancers in the Cr-added ZnO-based varistors. Some literature data are listed for comparison.^12,14,17−19,22−24,29,31−33^

Unlike previous reports, Cr is, in this work, found to be a varistor former rather than a performance enhancer for ZnO-based varistors. Cr was thought to be a performance enhancer and, in this role, together with other performance enhancers (i.e., Sb, Co, Mn, Ni, etc.) was added to ZnO along with the well-established varistor formers such as Bi and Pr.^17,19^ To verify whether Cr is a varistor former at the GB or a performance enhancer in the grains, a series of samples with nominal compositions Zn_1–*x*_Cr_2*x*_O_1+2*x*_ was synthesized. The X-ray powder-diffraction measurements (Figure S1) reveal that a pure hexagonal wurtzite structure is obtained when *x* ≤ 0.1%, above which a secondary phase, ZnCr_2_O_4_, forms. These arguments are corroborated by the backscattered electron (BSE) micrographs and the energy-dispersive X-ray spectroscopy (EDS) measurements (Figure S2). As shown in Figure 2a, no intergranular phases or amorphous regions are observed at the GB by high-resolution transmission electron microscopy (HRTEM). While no Cr is detected in the grains, within the EDS detection limit, for all of the samples (Figure S2) Cr is observed at the GB (inset of Figure 2a). Micro-Raman spectra also detect a strong vibration mode centered at ∼830 cm^–1^, which was previously reported in Cr-added ZnO nanocrystals,^26^ at the GB, but absent in the grains (Figure 2b). All of the data suggest a preference for Cr to stay at the GB instead of in the grain. Meanwhile, the oxygen content is higher while the Zn content is lower at the GB compared to within the grain, indicating that the segregation of the Cr at the GBs leads to an O-rich GB compared to the grain (Figure S3). In light of the case study of ZnO-Bi_2_O_3_-based and ZnO-Pr_6_O_11_-based varistors,^27,28^ the segregation of Cr at the GB leads to the formation of a Cr_Zn_ + V_Zn_ + O_i_ defect complex, which acts as a varistor former to shift the *E*_FB_ to a deeper position in the band gap to yield the DSBs and thus the nonlinear characteristics (Figure 2c).

The varistor former Cr elicits nonlinear *I–V* characteristics in ZnO, but the α value is only about 2 in the ZnO-0.1% Cr_2_O_3_ sample (Figure 2c). Introducing performance enhancers is thus a must to enlarge the difference between *E*_FB_ and *E*_FG_, i.e., φ_B_. To this end, the results of our study show that codoping with Ca, Co, and Sb acts as an effective performance enhancer in the presence of the varistor former Cr. Figure S4a, which shows elemental mapping, indicates that Ca, Co, and Sb are homogeneously distributed in the grains in the absence of Cr within the EDS detection limit. Hereafter, the chemical formula (Ca, Co, Sb)-doped ZnO with a varistor former derived from *x*% Cr_2_O_3_ will be written in the form (Ca, Co, Sb)-doped ZnO-*x*% Cr_2_O_3_.

Figure 2c shows that the nonlinear current density vs. electric field (*J–E*) characteristics are observed in all of the Cr-added ZnO ceramics, but the α values vary by several orders of magnitude with the specific compositions of the performance enhancers (Table 1). The detailed electrical properties (α, *I*_L_ and *E*_b_, shown in Tables S1–S3) of the ZnO ceramics with various Ca-, Co-, and Cr-doping ratios are presented in the Supporting Information. In the following, we will focus on the samples with 0.1 mol % Cr_2_O_3_, 2 mol % CaCO_3_, 0.5 mol % Co_3_O_4_, and 0.15 mol % Sb_2_O_3_ ratios as an illustration and for conciseness. As shown in Figure 2d and Table 1, the α value increases from about 2 for ZnO-0.1% Cr_2_O_3_, to 27 for Ca-doped ZnO-0.1% Cr_2_O_3_, to 73 for (Ca, Co)-codoped ZnO-0.1% Cr_2_O_3_, and finally to an exceptional value of 219 in (Ca, Co, Sb)-codoped ZnO-0.1% Cr_2_O_3_. It is not rare to observe increased α values with an increasing number of enhancers (dopants);^29^ however, the magnitude of the improvement attained here is unprecedented (Figure 2d). Importantly, the (Ca, Co, Sb)-codoped ZnO-0.1% Cr_2_O_3_ ceramic has a very low *I*_L_ of less than 0.2 μA/cm^2^, also superior to those of previous state-of-the-art varistors (Figure 1b).^12,16,18,19^ Furthermore, a high *E*_b_ of 925 V/mm is obtained in the (Ca, Co, Sb)-codoped ZnO-0.1% Cr_2_O_3_ ceramic due to the greatly decreased grain size (Figure S5).

**Table 1:** Electrical properties of Cr-added ZnO varistor ceramics with different performance enhancers

sample	α	*I*_L_ (μA/cm^2^)	*E*_b_ (V/mm)
ZnO-0.1%Cr_2_O_3_	2		
Ca-ZnO-0.1%Cr_2_O_3_	27	8.6	424
(Ca,Co)-ZnO-0.1%Cr_2_O_3_	73	<0.2	394
(Ca,Co,Sb)-ZnO-0.1%Cr_2_O_3_	219	<0.2	925

The very low *I*_L_ and greatly decreased grain size suggest a high GB resistance. This argument is corroborated by the results of impedance spectroscopy. As shown in Figure S6, the resistances of the GBs are much larger than those of the grains in all of the Cr-containing ZnO ceramics. In particular, the (Ca, Co, Sb)-codoped ZnO-0.1% Cr_2_O_3_ ceramic shows an extremely high resistance at the GBs, an indication of the as-formed DSB.^2,30^

**Figure 3:**
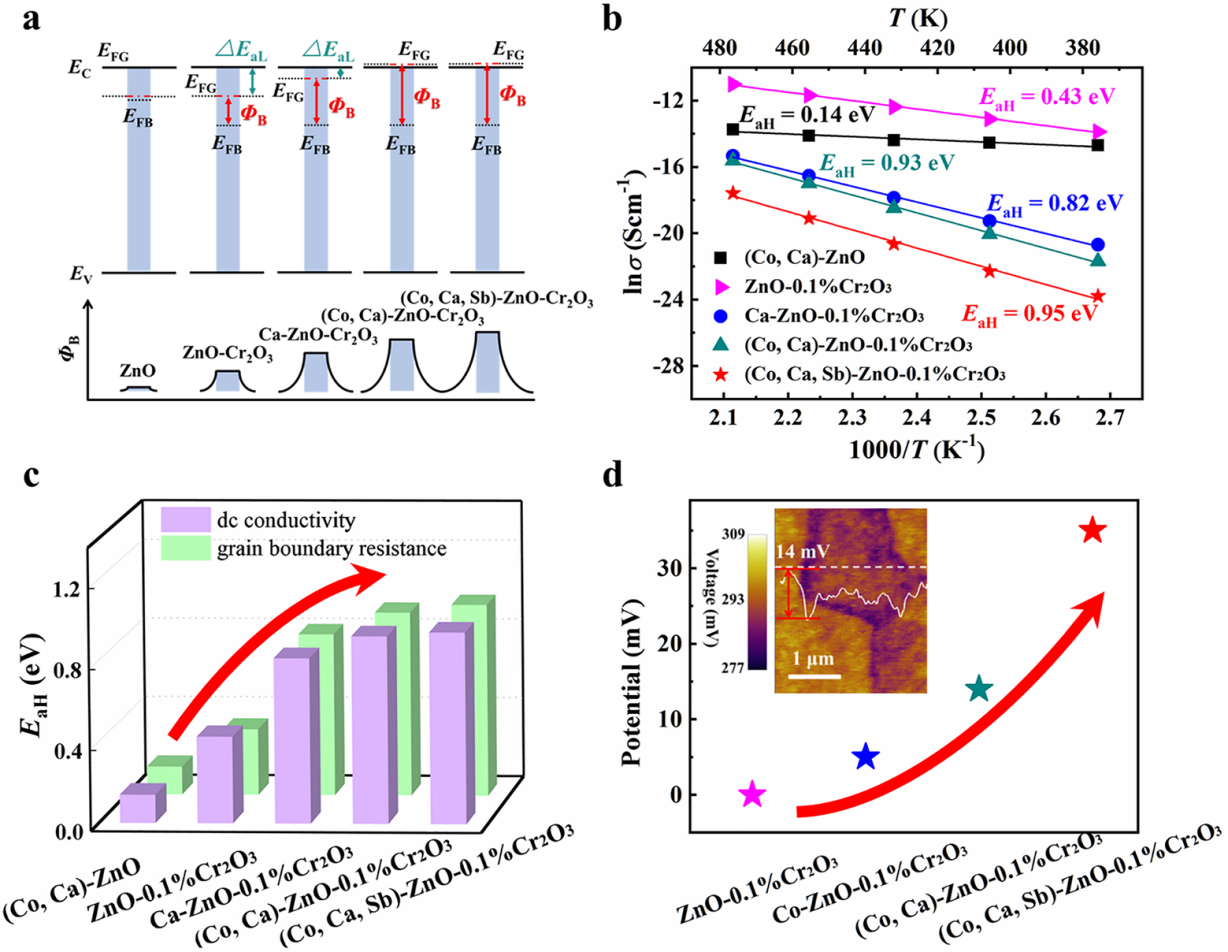
Evolution of the band structure, double-Schottky barrier height, high-temperature excitation energy, and surface potential of ZnO-based varistors with former Cr and enhancers Ca, Co, and Sb. (a) Schematic diagram of the DSB and band structure. (b) High-temperature dc conductivities vs. temperature. Solid lines are the fit to the Arrhenius model. Derived high-temperature activation energy *E*_aH_ values are labeled. (c) *E*_aH_ derived from the dc electrical conductivities (purple) and GB resistances (green). (d) Surface potentials. (Inset) Line-scan profile of the surface potential of (Co, Ca)-ZnO-0.1% Cr_2_O_3_.

The record-high varistor performance in 0.2% Cr-added (Co, Ca, Sb)-codoped ZnO ceramics with a high α value, a very low *I*_L_ value, and a high *E*_b_ value needs to be related to the band structure and the added performance enhancers (Figure 3a). As discussed above, *E*_FB_ is close to *E*_FG_ in the pristine and 0.2% Cr-added ZnO ceramics, yielding small α values. When adding the performance enhancers in ZnO, shallow donor levels form near the bottom of the conduction band of the grains, thus raising *E*_FG_, φ_B_, and α.

To probe the shallow donor levels in the grains, we performed temperature-dependent dc conductivity measurements. The activation energy (*E*_a_) is estimated by fitting the experimental data for the dc conductivity to the Arrhenius relation , where σ_0_ is a constant, *k*_B_ is the Boltzmann constant, and *T* is the absolute temperature. As shown in Figure 3b and Figure S7, the high-temperature σ data (373–473 K) and the low-temperature σ data (below 253 K) point toward two distinct *E*_a_ values, hereafter termed *E*_aL_ and *E*_aH_, respectively. With the composition varying from ZnO-0.1% Cr_2_O_3_, to Ca-doped ZnO-0.1% Cr_2_O_3_, to (Ca, Co)-doped ZnO-0.1% Cr_2_O_3_, and finally to (Ca, Co, Sb)-doped ZnO-0.1% Cr_2_O_3_, *E*_aL_ and *E*_aH_ evolve with opposite trends: *E*_aH_ systematically increases (cf. Figure 3b), whereas *E*_aL_ systematically decreases (Figure S7b). The decreasing *E*_aL_ indicates that the donor level becomes shallower in the band gap. Notably, the dc conductivities are nearly temperature independent at *T* < 253 K for (Co, Ca)-ZnO-0.1% Cr_2_O_3_ and (Co, Ca, Sb)-ZnO-0.1% Cr_2_O_3_, suggesting that the Fermi levels of the grain *E*_FG_ in these two compositions shift into the conduction band with practically zero activation energy. The *E*_FG_ value is enhanced with an increasing number of dopants (enhancers). The increasing *E*_FG_ value helps increase the φ_B_ and α values (Figure 2d) and substantiates the band-structure diagram plotted in Figure 3a. More supporting evidence will be provided in the following.

The high-temperature dc conductivities are known to be sensitive to φ_B_.^34^ The results, including the fitted high-temperature activation energy *E*_aH_, are presented in Figure 3b. The *E*_aH_ value is proportional to φ_B_, i.e., |*E*_FB_ – *E*_FG_|.^34^ The *E*_aH_ value of the (Co,Ca)-codoped ZnO ceramic is found to be 0.14 eV, similar to that of pristine ZnO, as shown in Figure S8. This implies that the *E*_FG_ and *E*_FB_ values are close, giving rise to a very weakly nonlinear *I*–*V* behavior (Figure S9). These results also indicate that Ca and Co are not effective varistor formers. In contrast, the *E*_aH_ value for the ZnO-1% Cr_2_O_3_ ceramic is 0.43 eV, significantly higher than that of ZnO and (Co, Ca)-doped ZnO. Thus, Cr is an effective varistor former. The *E*_aH_ value is further increased to 0.82 eV in Co-ZnO-0.1% Cr_2_O_3_, to 0.93 eV in (Co, Ca)-ZnO-0.1% Cr_2_O_3_, and to 0.95 eV in (Co, Ca, Sb)-ZnO-0.1% Cr_2_O_3_. The *E*_aH_ value can also be cross-checked by decomposing the high-temperature complex impedance Z* into the grain resistance *R*_g_ and the GB resistance *R*_b_ and fitting the temperature dependence of *R*_b_ into the Arrhenius model (Figure S10). As shown in Figure 3c, the *E*_aH_ values derived from the high-temperature dc conductivities and high-temperature impedance spectra agree well with each other.

All of the results presented and discussed in the last two paragraphs thus support the schematic diagram shown in Figure 3a, in which φ_B_ is significantly enhanced with an increasing number of dopants (enhancers). The systematically enhanced Schottky barrier height is further confirmed by the results from scanning Kelvin probe microscopy (Figure 3d and Figure S11). The surface potential across the GBs in ZnO-0.1% Cr_2_O_3_ is low and under the detection limit, but it quickly increases to about 5 mV in Ca-doped ZnO-0.1% Cr_2_O_3_, to 14 mV in (Co, Ca)-doped ZnO-0.1% Cr_2_O_3_, and finally to 35 mV in (Co, Ca, Sb)-ZnO-0.1% Cr_2_O_3_, consistent with the trend in the variation of *E*_aH_.

## Conclusions

A novel type of ZnO-based varistor ceramic with Cr as the varistor former and Ca, Co, and Sb as performance enhancers was discovered with simultaneously an ultrahigh nonlinear coefficient, a high breakdown voltage, and a low leakage current, superior to all of the state-of-the-art varistor ceramics, including the existing ZnO-based ones. The breakthrough is not only in the varistor’s performance but also in terms of the materials sustainability in terms of chemical stability, being environmental friendly, and having low cost. Specifically, the element Cr at the GBs as a varistor former and the proper performance enhancers (Ca, Co, Sb) doped in the grain work in tandem to yield a record-high varistor performance for the novel ZnO-Cr_2_O_3_ type of varistor ceramics.

The discovery of a novel ZnO-Cr_2_O_3_-type varistor ceramic represents the first such major breakthrough in the field of overvoltage protection after several decades that were dominated by the ZnO-Bi_2_O_3_-based ceramics. The results also provide insights into the design of the material and the development of nonlinear varistor ceramics with enhanced performance.

## Supporting Information Available

Additional characterization of Zn_1–*x*_Cr_2*x*_O_1+2*x*_, (Co, Ca, Sb)-ZnO-0.1% Cr_2_O_3_, Ca-ZnO-0.1% Cr_2_O_3_, and (Co, Ca)-ZnO-0.1% Cr_2_O_3_; additional electrical properties of ZnO-0.1% Cr_2_O_3_, Ca-ZnO-0.1% Cr_2_O_3_, (Co, Ca)-ZnO-0.1% Cr_2_O_3_, (Co, Ca, Sb)-ZnO-0.1% Cr_2_O_3_, ZnO, and (Co, Ca)-ZnO; surface potential images by AFM for (Co, Ca)-ZnO-0.1% Cr_2_O_3,_ (Co, Ca)-ZnO, and ZnO-Cr_2_O_3_ ceramics (PDF)
