# Supplementary material for: Novel Ultrahigh-Performance ZnO-Based Varistor Ceramics
Source: ACS Appl Mater Interfaces. 2021 Jul 23;13(30):35924–9. doi: 10.1021/acsami.1c07735 (PMC8397243; doi:10.1021/acsami.1c07735)
Supplement: Supplementary file 1 — am1c07735_si_001.pdf [file am1c07735_si_001.pdf]

Supporting Information

Novel ultra-high-performance ZnO-based varistor ceramics

Tian Tian,[1] Liaoying Zheng,[1] Matejka Podlogar,[3] Huarong Zeng,[1] Slavko Bernik,*[,3] Kunqi Xu,[1] Xuezheng Ruan,[1] Xun Shi,*[,2] Guorong Li*[,1]

1. *CAS Key Laboratory of Inorganic Functional Materials and Devices, Shanghai Institute of Ceramics, Chinese Academy of Sciences, Shanghai 201899, China*
2. *State Key Laboratory of High Performance Ceramics and Superfine, Microstructure, Shanghai Institute of Ceramics, Chinese Academy of Sciences, Shanghai 200050, China*
3. *Department for Nanostructured Materials, Jozef Stefan Institute, Ljubljana SI-1000, Slovenia*

E-mail: slavko.bernik@ijs.si; xshi@mail.sic.ac.cn; grli@mail.sic.ac.cn

Supplementary Text

A complex impedance measurement on polycrystalline solids can separate the specific contribution from the bulk (grain) and the internal surfaces (grain boundaries) to the total electrical conductivity. All the samples exhibit a single semi-circle within the measured frequency range, which is attributed to the response of the GB. Thus, only one constituent GB contributes to the measured complex impedance. The high-frequency (left-hand side of Figure S6a) and low-frequency (right-hand side of Figure S6a) intercept the real component axis (Z′axis) at R_g and $R_g + R_b$, respectively, where R_g is the resistance of the grain and R_b is the resistance of the grain boundary. The grain contribution to the total resistance is negligible compared to the grain-boundary contribution. The resistance of the (Co, Ca, Sb)-ZnO-0.1% Cr_2O_3 is increased by 2 orders of magnitude relative to that of the ZnO-0.1% Cr_2O_3.

We use one parallel RQ element to fit the impedance spectrum, which is shown in the inset of Figure S10a. Here, R_g and R_b represent the resistances of the grain and the GB, respectively, and Q_b is the corresponding phase element[1].

The formation of the potential barrier was confirmed with scanning Kelvin probe microscopy. The surface potential drop is clearly observed across the GBs for the (Co, Ca)-ZnO-0.1% Cr_2O_3 ceramic, which is not due to the crosstalk from topographical fluctuations (Figure S11a, b and d). In contrast, both the surface potential image (Figure S11d) and the enlarged one (Figure 3d) show no notable fluctuation in surface potential across the GBs for (Co, Ca)-ZnO.

Although there are some pores as shown in Figure S5, all the samples shows very high density (Table S4).

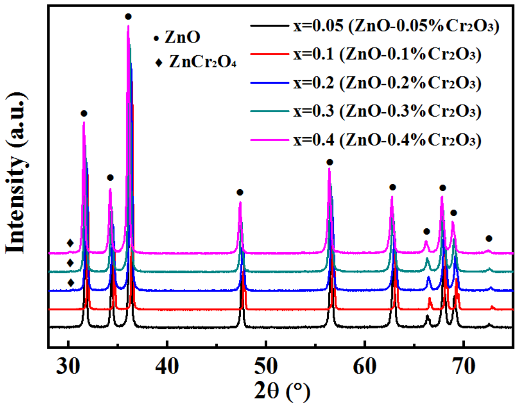

Figure S1. XRD patterns of $Zn_{1-x}Cr_{2x}O_{1+2x}$ (x = 0.05%, 0.1%, 0.2%, 0.3%, 0.4%). When x is equal to and higher than 0.2%, the $ZnCr_2O_4$ impurity phase is detected. Thus, the upper solubility limit for Cr in ZnO is between 0.1% and 0.2%.

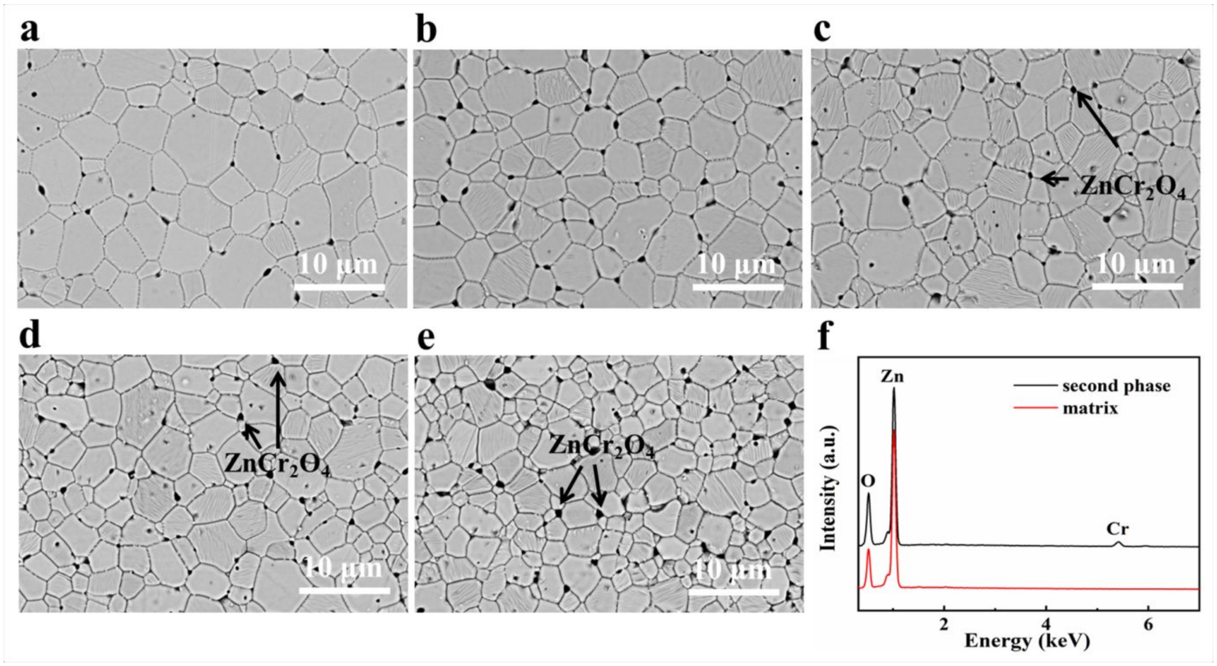

Figure S2. Microstructures of $Zn_{1-x}Cr_{2x}O_{1+2x}$. Backscattered-electron (BSE) images of $Zn_{1-x}Cr_{2x}O_{1+2x}$, x= a) 0.05%, b) 0.1%, c) 0.2%, d) 0.3%, e) 0.4%; f) Energy-dispersive X-ray spectroscopy (EDS) analyses for the matrix and the second phase of $Zn_{0.997}Cr_{0.006}O_{1.006}$. The EDS analyses for the matrix of $Zn_{1-x}Cr_{2x}O_{1+2x}$, x=0.05%, 0.1%, 0.2%, 0.4% and the second phase of $Zn_{1-x}Cr_{2x}O_{1+2x}$, x= 0.2%, 0.4% is similar to that of $Zn_{0.997}Cr_{0.006}O_{1.006}$ and is not shown in Figure S2. The black areas marked by the arrows is the $ZnCr_2O_4$ phase confirmed by EDS analysis.

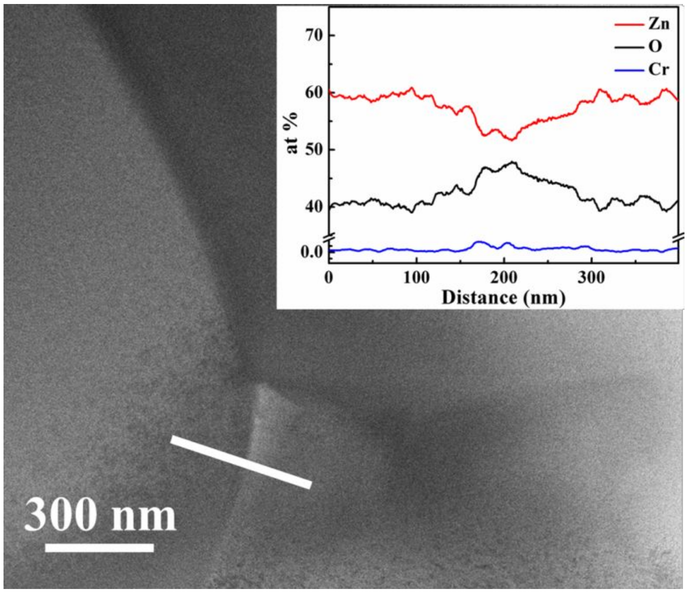

Figure S3. Scanning transmission electron microscopy (STEM) image of ZnO-0.1% Cr_2O_3. The inset is the EDS line-scan across a GB of ZnO-0.1% Cr_2O_3 as indicated by the white line.

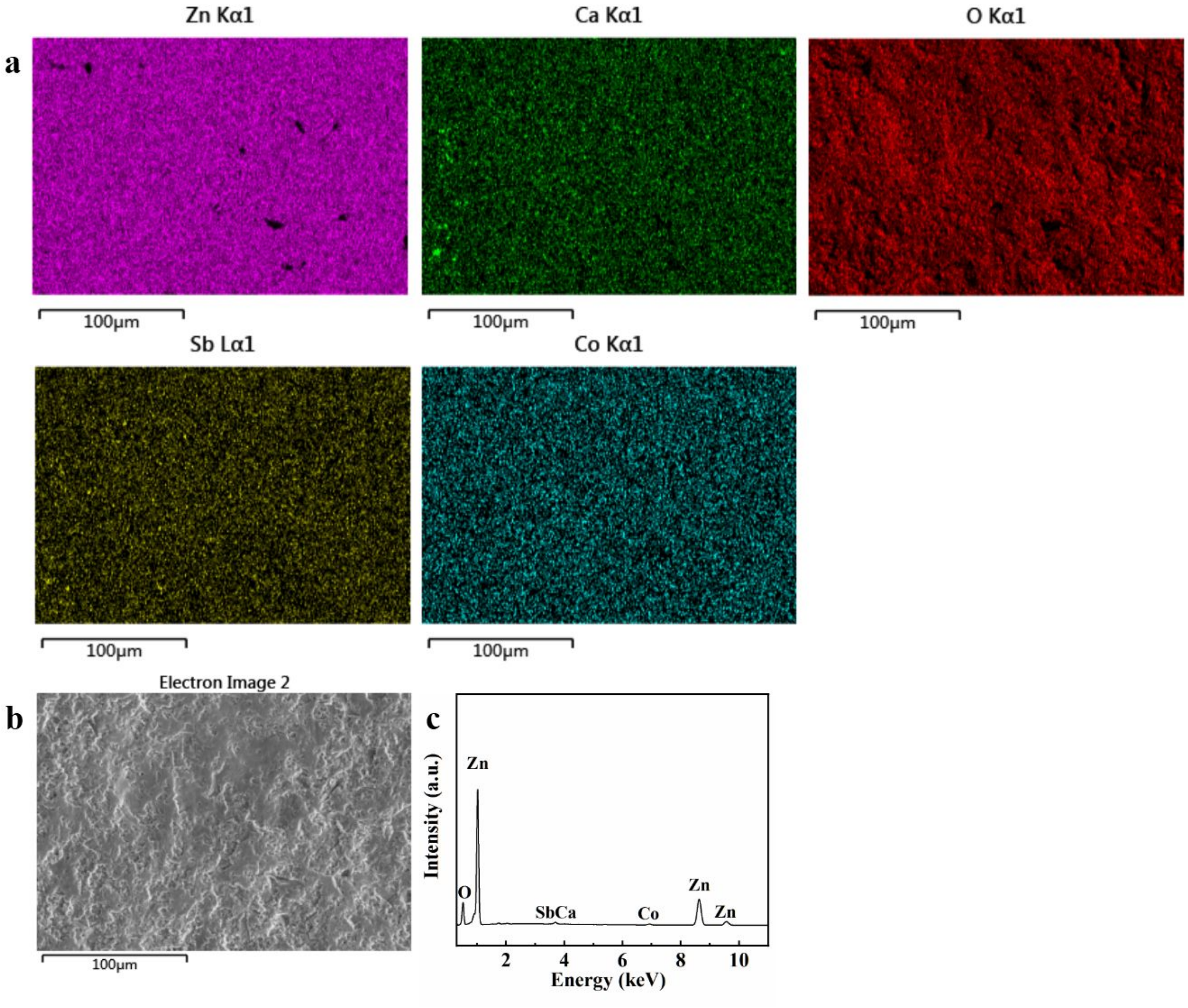

Figure S4. a) Elemental distributions, b) a Scanning Electron Microscope (SEM) image and c) an energy-dispersive X-ray spectroscopy (EDS) analysis of a grain of (Co, Ca, Sb)-ZnO-0.1% Cr_2O_3.

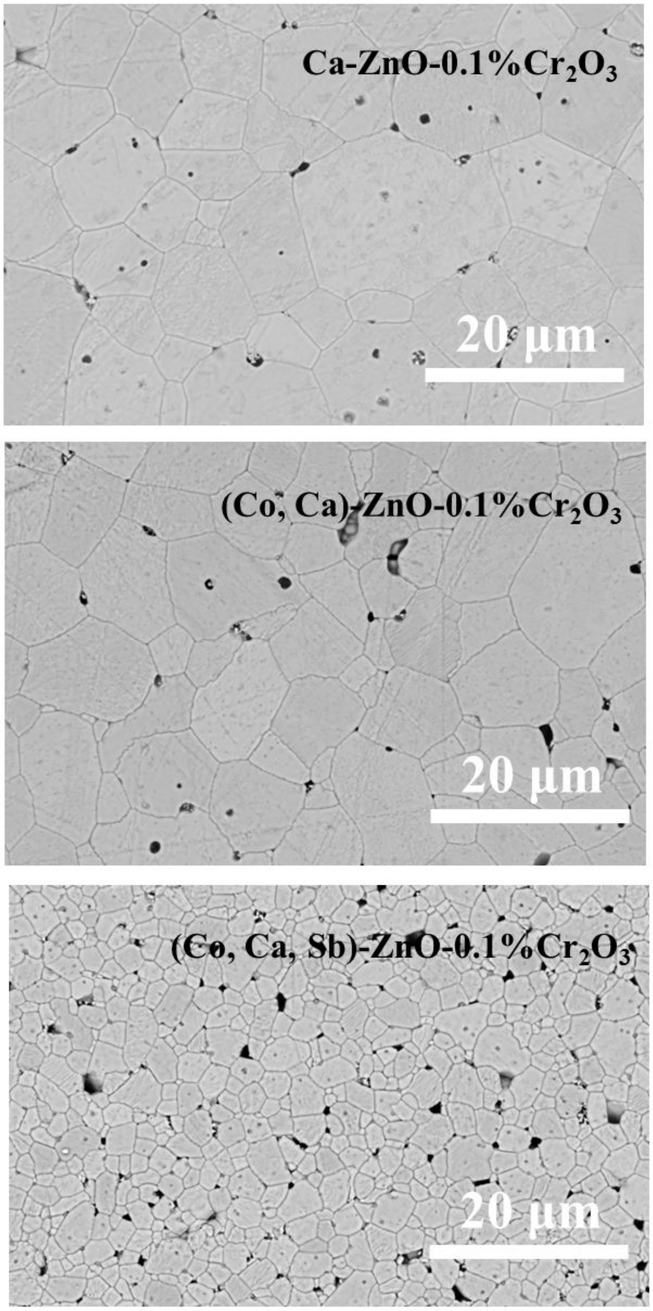

Figure S5. Back-scattered electron (BSE) images of Ca-ZnO-0.1% Cr_2O_3, (Co, Ca)-ZnO-0.1% Cr_2O_3 and (Co, Ca, Sb)-ZnO-0.1% Cr_2O_3.

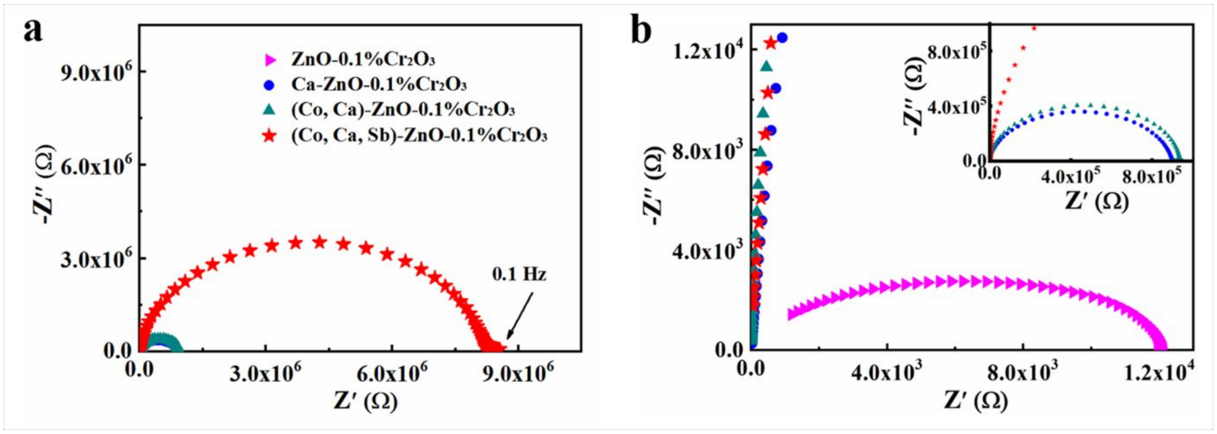

Figure S6. a) Complex impedance plots of ZnO-0.1% Cr_2O_3, Ca-ZnO-0.1% Cr_2O_3, (Co, Ca)-ZnO-0.1% Cr_2O_3 and (Co, Ca, Sb)-ZnO-0.1% Cr_2O_3 at 200℃. b) Complex impedance data in Figure S6a on small scales, and the inset is an intermediate scale image.

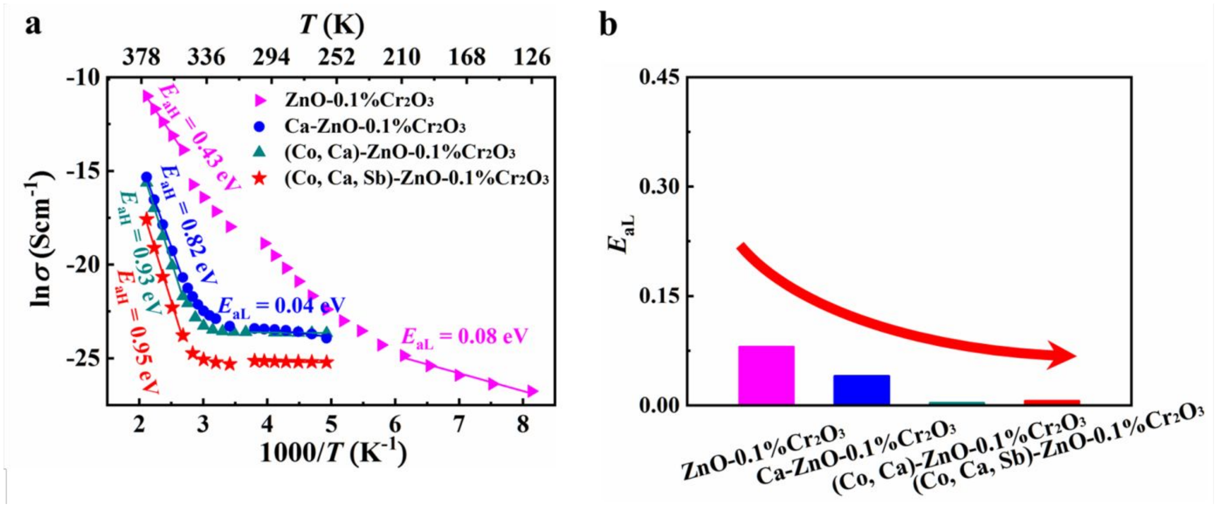

Figure S7. a) Conductivities versus temperature for Cr-added ZnO-based varistors. Solid lines are the fitted curves using the Arrhenius model. b) The activation energy (E_{aL}) of Cr-added ZnO-based varistors.

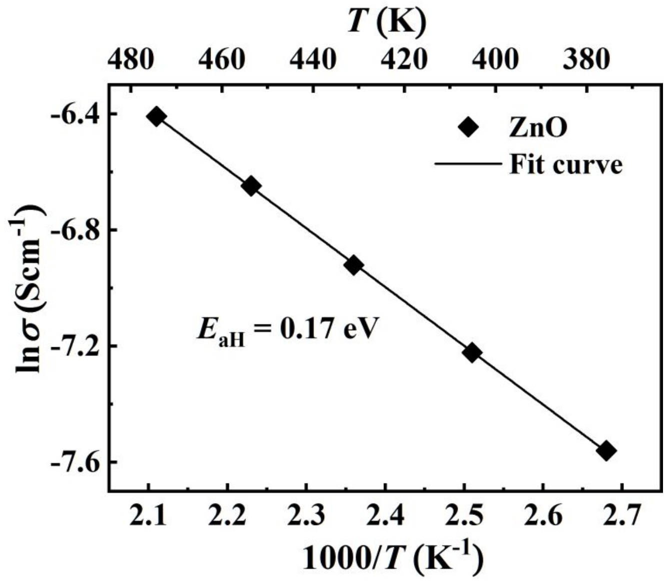

Figure S8. DC conductivities versus temperature for ZnO. The solid line is the fitted curves using the Arrhenius model.

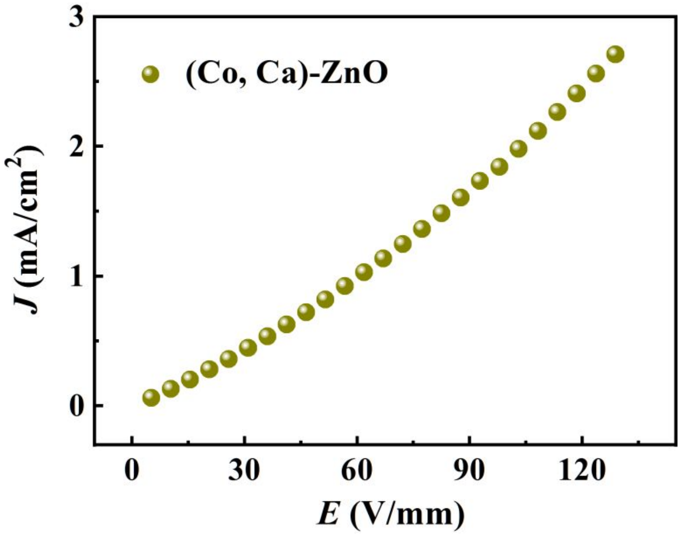

Figure S9. The *J*–*E* characteristic of (Co, Ca)-ZnO.

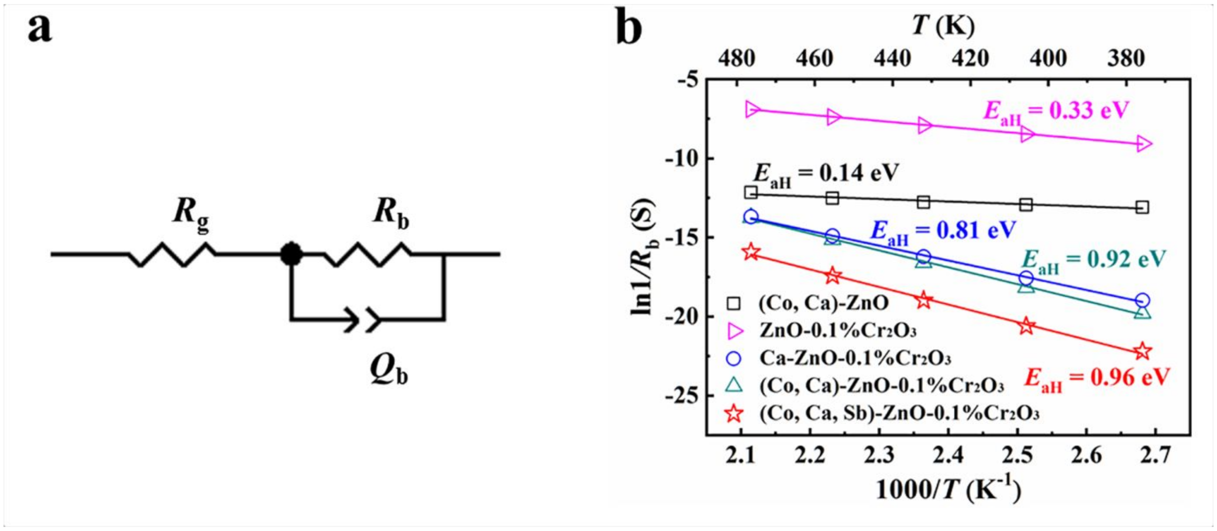

Figure S10. a) The equivalent circuit and b) grain-boundary resistances versus temperature for Cr-added ZnO-based varistors. The solid lines are the fitted curves using the Arrhenius model.

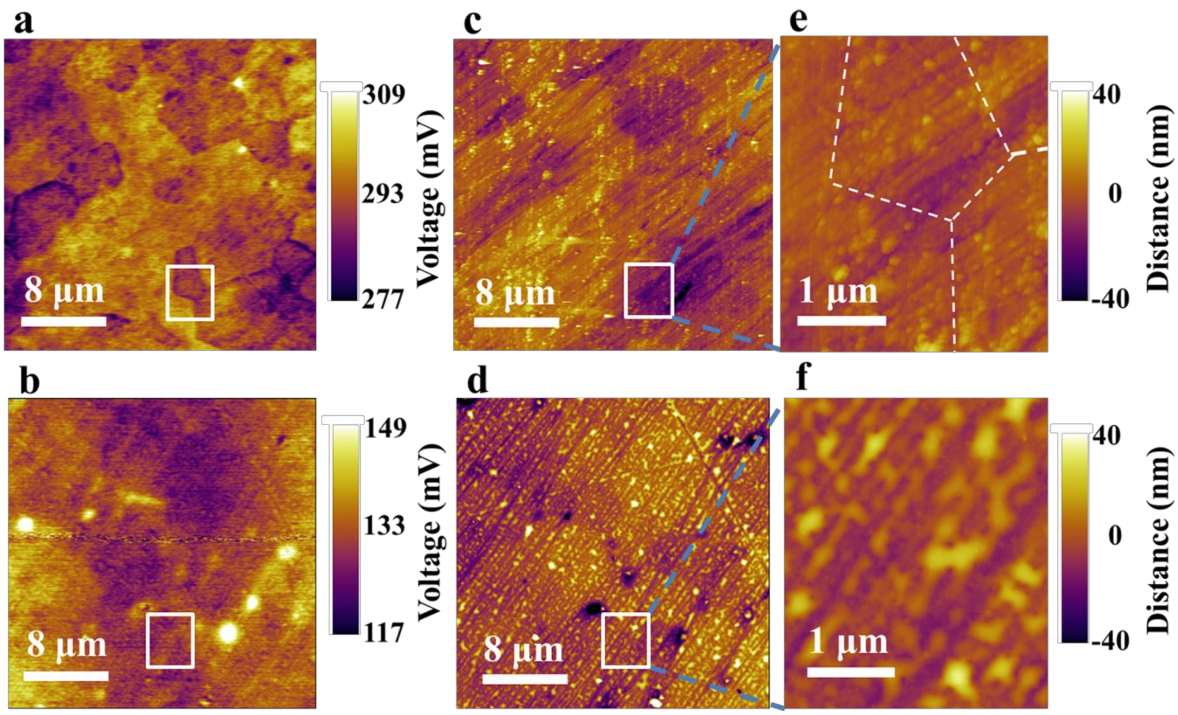

Figure S11. Surface potential images by atomic force microscopy (AFM) for a) (Co, Ca)-ZnO-0.1% Cr_2O_3 and b) (Co, Ca)-ZnO. The scanned region is 30 μm × 30 μm. c, d) are the surface topographic images of the same region scanned in (a) and (b). e, f) are in the enlarged pictures in the square regions of (c) and (d).

Table S1. Electrical properties of (Ca, Co, La)-ZnO-0.1% Cr_2O_3 ceramics with different amounts of Ca. The nominal compositions are (99.47-x) mol% ZnO + x mol% $CaCO_3$ + 0.33 mol %Co_3O_4 + 0.1 mol% Cr_2O_3 +0.1mol.% La_2O_3, and with x ranging from 0 to 6 (0, 0.5, 1, 2, 3, 4, 6).

$CaCO_3$ (mol%)	α	I_L (μA/cm^2)	E_b (V/mm)
0	17	21.9	877
0.5	24	3.5	857
1	30	1.1	579
2	40	2.8	520
3	28	2.5	539
4	29	2.1	616
6	27	1.8	600

Table S2. Electrical properties of (Ca, Co)-ZnO-0.1% Cr_2O_3 ceramics with different amounts of Co. The nominal compositions are (97.9-x) mol% ZnO + x mol% Co_3O_4 + 2 mol% $CaCO_3$ + 0.1 mol% Cr_2O_3, and with x ranging from 0 to 1 (0, 0.3, 0.5, 0.75, 1).

Co_3O_4 (mol%)	α	I_L (μA/cm^2)	E_b (V/mm)
0	27	8.6	424
0.3	47	$<$0.2	335
0.5	73	$<$0.2	394
0.75	54	0.31	428
1	39	0.31	412

Table S3. Electrical properties of (Ca, Co)-ZnO-xCr_2O_3 ceramics. The nominal compositions are (97.5-x) mol% ZnO + 0.5 mol% Co_3O_4 + 2 mol% $CaCO_3$ + x mol% Cr_2O_3, and with x ranging from 0.05 to 0.6 (0.05, 0.1, 0.2, 0.4, 0.6).

Cr_2O_3 (mol%)	α	I_L (μA/cm^2)	E_b (V/mm)
0.05	38	0.2	341
0.1	73	<0.2	394
0.2	54	<0.2	291
0.4	45	10.7	285
0.6	25	5.8	352

Table S4. Sintered density for the Cr-added ZnO-based varistors.

Sample	Sintered density (g/cm^3)	Relative density
ZnO-0.1% Cr_2O_3	5.24	94%
Ca- ZnO-0.1% Cr_2O_3	5.34	95%
(Ca, Co)-ZnO-0.1% Cr_2O_3	5.42	97%
(Ca, Co, Sb)-ZnO-0.1% Cr_2O_3	5.36	96%

References

1. Haile, S. M.; West, D. L.; Campbell, J. The Role of Microstructure and Processing on the Proton Conducting Properties of Gadolinium-doped Barium Cerate. *J. Mater. Res.* **1998**, 13, 1576.
